# Protective Effect of Escin Against Kidney Injury: Histopathological and Biochemical Evaluations

**DOI:** 10.3390/cimb46120804

**Published:** 2024-11-25

**Authors:** Mustafa Cengiz, Betül Peker Cengiz, Alanna Teixeira Andrade, Adnan Ayhanci

**Affiliations:** 1 Department of Elementary Education, Siirt University, 561000 Siirt, Türkiye; 2 Department of Pathology, Eskişehir Yunus Emre State Hospital, 26130 Eskişehir, Türkiye; 3 Department of Biology, Eskisehir Osmangazi University, 26000 Eskişehir, Türkiyeaayhanci@ogu.edu.tr (A.A.)

**Keywords:** escin, cyclophosphamide, histological alternations, oxidative stress

## Abstract

The purpose of the current study was to find out whether escin (ES) safeguarded experimental rats against cyclophosphamide (CP)-induced kidney injury. Twenty-four rats were randomly divided into four groups (*n* = 6). After the examination, histological and biochemical analyses were performed to assess the alterations in kidney tissue. According to histologic and biochemical analyses, renal tissue in the CP group suffered significant damage by CP. There was a significant improvement in histological damage in the group receiving CP+ES together. This suggests that ES significantly protects the kidney’s functional characteristics. The present study concludes by highlighting histological and biochemical studies to illustrate the ability of ES to cure kidney injury caused by CP and its influence on the relationship between oxidative stress, apoptosis, and renal failure.

## 1. Introduction

Many harmful xenobiotic compounds, such as medicines, metals, and environmental toxins, have a high affinity for the kidney, thus making it a prime target organ [1,2]. Drug-induced kidney damage has many pathological consequences and is not an uncommon adverse effect [3,4]. Cyclophosphamide (CP), well known as an anticancer drug, is used to treat a variety of cancers, including breast, lung, and ovarian cancers, as well as other malignancies, including lymphoma. Its harmful effects on organs including the kidney, liver, brain, and bladder, however, limit its usage [5,6,7]. Previous studies have shown that CP-induced kidney damage is caused by increased oxidative stress (OS) [8,9,10,11,12]. It is reasonable to consider using antioxidant agents with different protective and detoxifying properties that scavenge free radicals to prevent or significantly reduce the harmful effects of CP [11,13,14,15].

Thiols, also known as mercaptans, are chemical molecules with a sulfhydryl (-SH) group that bind mercury. The -SH group found in thiols protects against not just oxidative damage but also apoptosis. During the interaction between thiols and oxygen radicals, reversible disulfide bonds develop. Recently, thiol–disulfide homeostasis has been recognized as an indicator of OS. The achievement of thiol–disulfide equilibrium is attributed to the dissociation of disulfide bonds from thiol groups, resulting in thiol–disulfide equilibrium [16,17].

ES is a naturally occurring blend of pentacyclic triterpenoid saponins that were separated from *Aesculus hippocastanum* and *Aesculus indica* [18]. Recent research, however, has found it to have anti-inflammatory, antioxidant, anti-edematous, antiviral, and anti-tumoral properties [19]. Escin has a trisaccharide (glucose, xylose, and galactose) attached to a 3-OH residue. An organic acid (such as acetic, tiglinic, or angelic acid) is used to esterify the C21 and C22 domains. Kryptoescin and β-escin are the primary escin isomers, and they serve as the foundation for the pharmacological preparations of escin formulations in this study. In contrast to β-escin, which is comparatively insoluble in water, kryptoescin is easily soluble in water but has a much lower level of activity. Escin has a molecular weight of 1131.27 Da and the chemical formula C_55_H_86_O_24_ [20]. No reports evaluating the protective effects of ES against CP-induced kidney injury were found in the literature. This study sought to ascertain if ES might shield experimental rats against oxidative stress, apoptosis, and histopathologic changes by CP.

## 2. Materials and Methods

### 2.1. Chemicals and Drugs

CP (98% purity; CAS number: 6055-19-2) and 99% pure ES (CAS number: 6805-41-0; PHL80446-Escin IB) were acquired from trading firms (Sigma-Aldrich, Darmstadt, Ger-many). The kits that remained were acquired from commercial sources.

### 2.2. Animal Handling and Experimental Design

Twenty-four male Sprague Dawley rats (3–4 month), weighing 180–240 g, were used in this study (four groups, *n* = 6). Rats acquired from the Experimental Animal Center were kept in a regular habitat (45–50% humidity, 22 ± 2 °C, 12 h of sunshine, and 12 h of darkness) and provided drinking water and standard pellets. Only after receiving ethical approval from the Animal Welfare Committee at Eskisehir Osmangazi University (Ethics Committee Number: 2018/670-1) were all the animal treatments completed.

### 2.3. Treatment of Experimental Animals

The rats received this amount of CP intravenously using sterilized disposable injectors; for the oral administration solution, 10 mg/kg ES [21] was dissolved in 0.5 mL of saline [22]. After receiving 0.5 mL of saline on the first day and being sacrificed the following day, the rats in the control group, CP, ES, and CP+ES groups received injections on the first day and were sacrificed the following day. Blood samples were taken by cardiac puncture under ether anesthesia, and the rats were killed the following day (Table 1).

### 2.4. Analysis of Renal Histopathology

For the histopathologic examination, kidney tissues were kept in 10% formalin solution for 48 h and then passed through alcohol and xylene series and embedded in paraffin blocks. Sections of 5 μm thickness were taken from each paraffin block, placed on a normal slide, and stained with hematoxylin and eosin (H&E). Pathological changes were then visualized under a microscope by a pathologist blinded to the animal treatments. The stained slides were examined and scored by light microscopy. The renal injury score for pathological lesions was based on an examination of ten randomly selected and non-overlapping areas for cortex and renal tubular tissues, and the percentage of damaged area was calculated according to the following criteria: tubular dilatation, intratubular cast formation, area of infiltration of inflammatory cells, and tubular cell necrosis.

Also, caspase-3, Bcl-2, and Bax, which are indicators of apoptosis, were examined using standard immunohistochemical methods [6]. With only little adjustments, immunostaining was accomplished in accordance with the conventional techniques. The testes sections were deparaffinized in xylene and rehydrated with the help of graded ethanol solutions after being fixed overnight at 36 °C. To quench endogenous peroxidase, the specimens were immersed in a freshly prepared solution of 1% H_2_O_2_ in ice-cold methanol for 20 min. These specimens were incubated for one hour at room temperature in blocking solution (3% BSA, 0.1% Tween-20 in PBS) after being able to be rinsed in phosphate-buffered saline (PBS). Anti-Bcl-2, anti-Bax, and anti-caspase-3 antibodies (goat polyclonal) (1:500) were used in blocking solution for 12 h at 4 °C. After being re-equilibrated to room temperature and cleaned with PBS, horse radish peroxidase (HRP) antibody conjugates (1:2500) were added to blocking solution without Tween-20 and incubated for 2 h at room temperature. Before being exposed to washing in distilled water, the specimens were cleaned with PBS and incubated with a 0.2% solution of 3, 3′-diaminobenzidine (DAB) until the required stain intensity was reached at room temperature. Hematoxylin was used as a counterstain, and di-n-butylphthalate–polystyrenexylene (DPX) was used to mount these sections. Three impartial observers blindly assessed immunoreactivity by counting the total number of cells that were positively stained. To determine the intensity of the positive expression, the number of positive cells in each high-power field was counted, and the average number of positive cells was computed. Based on staining positivity, sections were rated as none (0), weak (1), moderate (2), and severe (3).

### 2.5. Biochemical Analysis

#### 2.5.1. Determination of BUN and Cre Levels

The blood samples were centrifuged at 3000 rpm for ten minutes to obtain the serum of the test animals. The Cre and BUN levels, which are markers of kidney damage, in the serum were measured using an automatic biochemical autoanalyzer (HITACHI-917, Roche, Basel, Switzerland).

#### 2.5.2. Estimation of TT, SDS, and NT Levels

Analysis of serum thiol–disulfide profiles, as a marker of oxidative stress, was carried out with a Roche (Cobas 501, Roche, Basel, Switzerland), device. Furthermore, the comparison test was used to determine the TT and NT values. NT concentrations were subtracted from TT concentrations to determine the quantities of sulfur (SDS) (S-S). Consequently, the ratios of SDS/TT, SDS/NT, and NT/TT were computed [23].

### 2.6. Statistical Analyses

Using the standard error of the mean (±SEM), the expression of the data from animal experiments was determined. The analysis of normally distributed continuous data and independent measures was carried out using a one-way ANOVA, using SPSS program version 20 (SPSS, Richmond, VA, USA). Moreover, variants with aberrant distributions were scored using the Kruskal–Wallis test. *p* values <0.001, <0.01, and <0.05 were deemed significant for differences in the experimental groups.

## 3. Results

**Figure 1:**
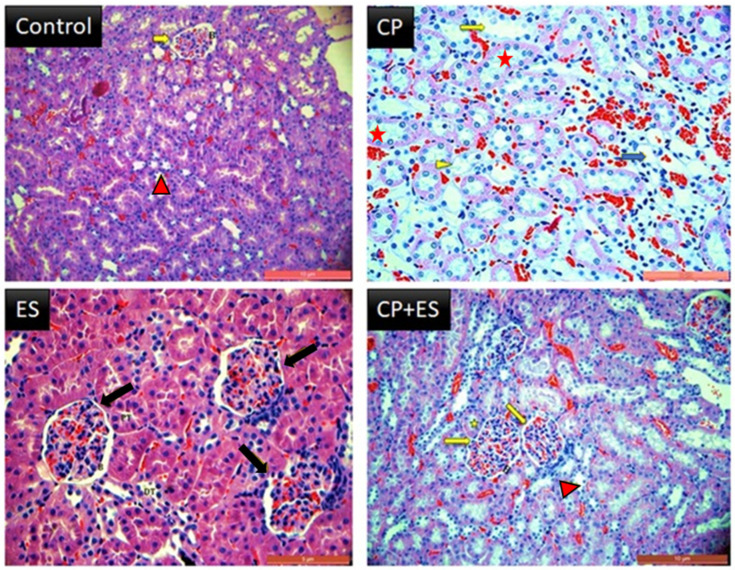
Kidney sections of all the study groups (H&E). Intact glomerulus (yellow arrow), a typical glomerular structure (yellow arrow and black arrow), a noticeable constriction of the bowman gap (blue arrow). Degenerated tubules (red and yellow arrowhead), and a small number of inflam-matory cell infiltrations (red and yellow star) (X20, X40).

The kidney sections of the control and ES groups showed a typical glomerular structure (yellow arrow and black arrow), thin capsule region, proximal convoluted tubules, and distal convoluted tubule epithelial cells. The control group, on the other hand, had little congestion, but the ES group had a limited number of inflammatory cell infiltrations. Such an unexpected result could be explained by the probability that the tissues were harmed over the follow-up period. The administration of CP was shown to result in degeneration, significant tubule congestion (star), inflammatory cell infiltration (yellow arrow, and black arrow), aberrant glomerulus structure, and a noticeable constriction of the bowman gap (blue arrow). Compared to the CP group, there was a significant improvement in histologic damage in the CP+ES group. This shift implies that ES could play a part in reducing the renal damage brought on by CP (Figure 1 and Table 2).

**Figure 2:**
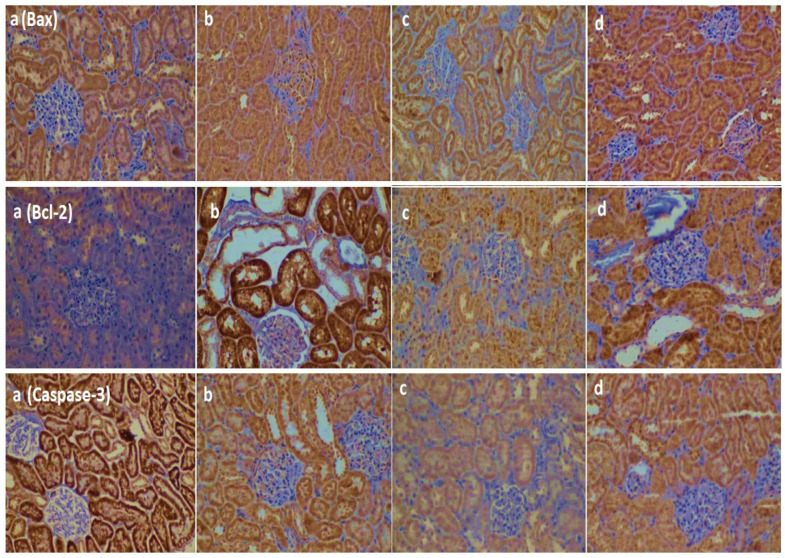
Bax (**a**–**d**), Bcl-2 (**a**–**d**), and caspase-3 (**a**–**d**) expression in all the study groups (X20).

As seen in Figure 2, a small number of Bax and caspase-3-positive cells were observed in the sections of the control and ES groups. The number of Bcl-2-positive cells was statistically significant (*p* < 0.05). In the CP group, the number of stained caspase-3- and Bax-positive cells was significantly higher, but the number of Bcl-2-positive cells was significantly lower (*p* < 0.05). When ES was administered together with CP, CP-induced apoptosis was significantly reduced by ES administration (Figure 2 and Table 3).

**Figure 3:**
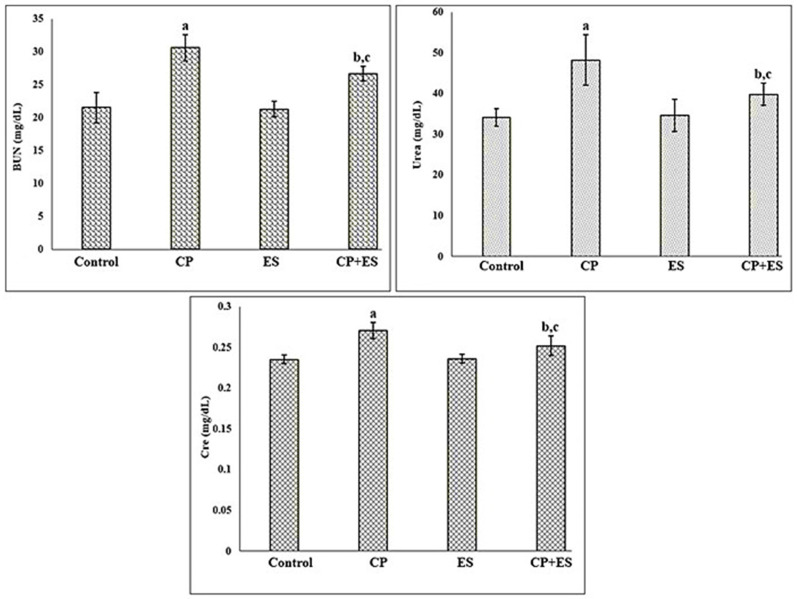
The BUN, Cre, and urea levels of all experimental rats (±SEM). ^a^ *p* < 0.05 and ^b^ *p* < 0.05 compared to control; ^c^ *p* < 0.05 compared to CP.

Serum BUN levels were significantly increased in the CP group (30.58 ± 1.98), which was statistically significant compared to the ES (21.25 ± 1.15) and control (21.50 ± 2.29) groups (*p* < 0.05), but significantly decreased in the group given ES with CP (26.62 ± 1.13) compared to the group given CP alone (*p* < 0.05). These findings suggest that ES attenuates CP-induced nephrotoxicity (Figure 3).

While serum urea levels were statistically significant higher in the CP group (48.21 ± 6.12) than in the ES (34.66 ± 3.96) and control (34.05 ± 2.17) groups (*p* < 0.05), they were considerably lower in the ES with CP group (39.76 ± 2.75) than in the CP alone group (*p* < 0.05). These results imply that ES reduces the nephrotoxicity brought on by CP (Figure 3).

Serum creatinine levels were statistically significantly elevated in the CP group (0.27 ± 0.009) compared to the ES (0.236 ± 0.005) and control (0.235 ± 0.005) groups (*p* < 0.05). Conversely, levels in the ES with CP group (0.251 ± 0.01) were significantly lower than those in the CP alone group (*p* < 0.05). As shown in Figure 3, the results indicate that ES mitigates the nephrotoxicity induced by CP (Figure 3).

**Figure 4:**
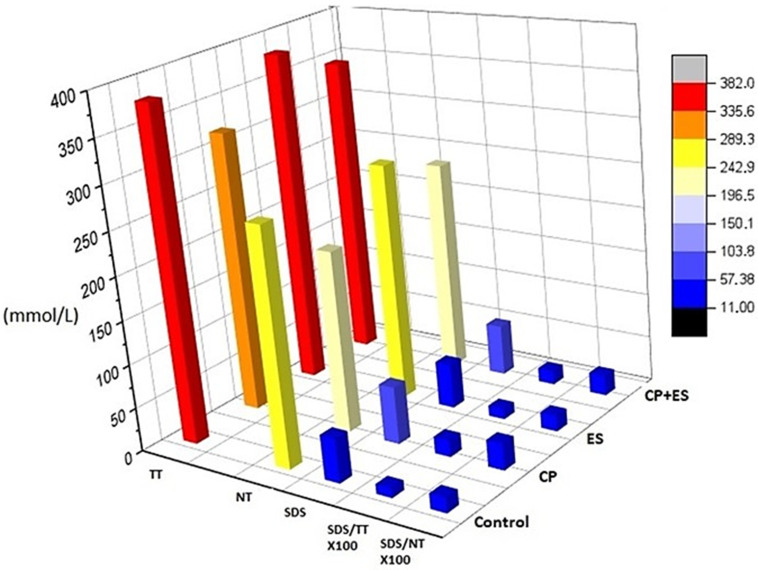
Serum TT, NT, and SDS levels (mmol/L) in the experimental groups.

Figure 4 displays the oxidative stress analysis findings. While the NT and TT levels were considerable in the control and ES groups, they significantly declined in the CP group (p < 0.05). Compared to the CP group, the NT and TT levels improved statistically significantly when ES and CP were administered together (p < 0.05). CP significantly increased the SDS levels compared to the other groups (control and ES) (p < 0.05). The SDS levels obtained when CP+ES was applied together were similar to the values of the other groups (SDS levels obtained when CP+ES was applied together were similar to the values of the other groups (control and ES)). A comparison of all groups in terms of the SDS/NT and SDS/TT levels revealed a statistically significant difference in the CP group compared to the other groups (control and ES) (p < 0.05). No statistically significant difference in the SDS/NT levels was observed in the CP+ES group compared to the control and ES groups. These findings suggest that ES may attenuate CP-induced oxidative stress.

## 4. Discussion

The kidney is recognized as a primary target for various xenobiotic toxicants, including pharmaceuticals, due to several factors that contribute to its heightened sensitivity. This sensitivity is linked to the abundance of metabolizing enzymes and xenobiotic transporters within the kidney, as well as its substantial blood flow. Additionally, the kidney’s unique physiological, anatomical, and biochemical properties increase its vulnerability to a range of toxins and medications. This study was conducted to investigate the potential role of ES in mitigating in vivo AKI induced by CP, which produces two active metabolites: phosphoramide and acrolein [24,25]. Phosphoramide exhibits anticancer activity, whereas acrolein can induce nephrotoxicity, promote lipid peroxidation, and cause structural alterations in tissue, alongside a reduction in antioxidant activity [26,27,28]. Research has demonstrated that CP administration induces oxidative stress and apoptosis in various tissues of mice and rats [29]. The balance of dynamic thiol/disulfide homeostasis significantly influences antioxidant defenses, apoptotic processes, detoxification mechanisms, and the regulation of enzymatic activities [16].

Assessing oxidative stress through thiol/disulfide parameters using a novel method developed by Erel et al. has recently garnered global interest as it effectively reflects the organism’s oxidation and total thiol (TT) capacity [16]. Our previous study demonstrated that a 200 mg/kg dose of CP led to decreased serum NT and TT levels, alongside elevated levels of disulfide (SDS) [30]. It is noteworthy that no scientific publications currently exist in the literature regarding CP-induced changes in thiol/disulfide parameters. In this study, NT and TT levels in the CP group were reduced, while SDS levels were elevated.

Beyond inducing oxidative stress, CP treatment disrupts the balance of apoptosis-regulating proteins, including Bcl-2, Bax, and caspase-3. Apoptosis is tightly regulated by the Bcl-2 protein [31]. Increased expression of the anti-apoptotic protein Bcl-2 can help mitigate kidney injury, while elevated Bax expression promotes cell death. Bcl-2 also inhibits apoptosis by blocking mitochondrial cytochrome C release. Excessive reactive oxygen species can induce cell death by inhibiting Bcl-2 and activating Bax along with the mitochondrial apoptosis pathways. According to the immunohistochemical analysis reported in this study, the CP-treated group showed a reduced expression of Bcl-2 and elevated expression of Bax and caspase-3 (Figure 2), aligning with the findings from Liu et al. [32], Casteels et al. [33], and AlHaithloul et al. [34].

The significant elevation in serum BUN, urea, and creatinine levels serves as an indicator of nephrotoxicity. The CP-induced increases in these serum markers observed in this study are consistent with the findings reported by Rehman et al. [35]. Our histopathological results further support these biochemical parameters, as shown in the photomicrographs. Kidney tissues from CP-treated rats (Figure 1) exhibit structural abnormalities in the glomerulus, evident narrowing of Bowman’s space, degeneration of tubules, pronounced congestion, inflammatory cell infiltration, and tubular necrosis. These findings are consistent with previous studies by Rehman et al. [35], AlHaithloul et al. [34], and Sinanoglu et al. [36].

ES, a primary extract of Aesculus hippocastanum, is known for its anti-inflammatory, anti-edematous, and antioxidant properties [37,38,39]. Studies indicate that ES has protective effects against experimentally induced liver and lung injury as well as kidney damage associated with diabetes [37,38,40,41,42]. Salama and Elmalt [43] reported that in potassium dichromate (PD)-induced kidney injury, escin improved renal function, attenuated inflammatory and proliferative markers, and ameliorated hyaline materials in Bowman’s cavity and necrosis of proximal convoluted tubules. Moreover, serum creatinine and BUN levels were reported to increase by 76% and 87%, respectively, compared to the normal control group after PD-induced kidney injury. It was emphasized that pretreatment with escin decreased serum creatinine and BUN levels by 32% and 31%, respectively, while post-treatment serum creatinine and BUN levels decreased by 28% and 10%, respectively, compared to the PD group. In another study, escin was reported to alleviate peripheral neuropathy in streptozotocin-induced diabetes in rats by preventing oxidative stress [44]. However, to date, no studies have examined the effects of ES on CP-induced kidney injury. This study; therefore, is among the first to investigate the impact of ES on CP-induced kidney injury.

**Scheme 1:**
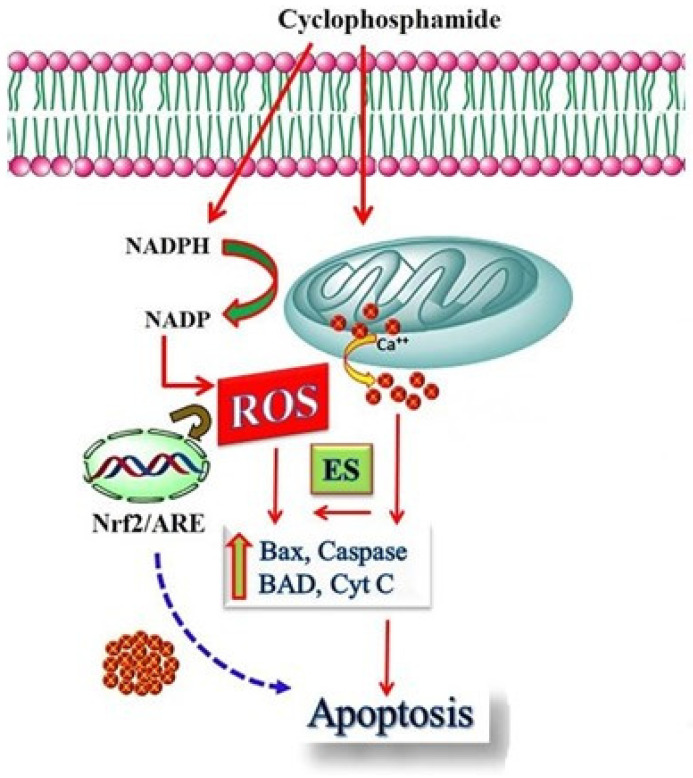
Possible protective mechanism of ES in CP-induced injury [45].

In conclusion, the biochemical, immunohistochemical, and histopathological results show that ES significantly reduces CP-induced oxidative stress, apoptosis, and tissue damage (Figure 1, Figure 2, Figure 3 and Figure 4 and Table 1). Based on our study results, we are of the opinion that the protective effects of ES upon CP-induced nephrotoxicity can be accounted for by its antioxidant properties. It is tempting to speculate that ES shows this protective effect by providing cellular membrane integrity and inhibiting ROS-induced apoptotic pathways (Scheme 1). Therefore, further studies are needed that seek to investigate the molecular pathways involved in the modulatory action of ES upon CP-induced kidney injury. Our study suggests that the antioxidant properties of ES may be attributed to its protective effects against CP-induced damage. Presumably, the ES seems to have a protective effect against cell damage caused by CP. ES appears to work to prevent or lessen the apoptotic process (Bax, caspase, BAD, Cyt C) by reducing the negative effects of ROS (see Scheme 1). Consequently, additional research is required to explore the molecular pathways involved in the modulatory effects of ES on CP-induced kidney damage.

## 5. Conclusions

Our results indicate that because ES may lower oxidative stress, tissue damage, and apoptosis, it may offer substantial therapeutic advantages against renal consequences of CP chemotherapy. Thus, from a therapeutic standpoint, our study indicates that ES’s protective benefits against CP-induced nephrotoxicity, in addition to its antineoplastic qualities documented in a number of malignancies, are very encouraging. To determine precisely the mechanism of ES action, more research is necessary.

## Figures and Tables

**Table 1:** Rat groups and period of the experiment schedule.

Group (*n* = 6)	Period of the Experiment
Control	0.5 mL saline, i.p. once on 1st day
CP	200 mg/kg, i.p. once on 1st day
ES	10 mg/kg, orally, once on 1st day
CP+ES	200 mg/kg, i.p. once on 1st day + 10 mg/kg, orally, once on 1st day

**Table 2:** Histological scoring.

Groups	Narrowing in Bowman	Tubular Damage	Inflammation	Glomerular Damage	Congestion	Total Score
Control	0	0	0	0	1	1
CP	3	3	1	2	3	12 ^a^
ES	0	0	1	0	0	1
CP+ES	1	1	1	1	1	5 ^b,c^

^a^ *p* < 0.05 and ^b^ *p* < 0.05 compared to control; ^c^ *p* < 0.05 compared to CP.

**Table 3:** Immunohistochemical results and their scores in kidney tissue.

Groups	Bax	Bcl-2	Caspase-3
Control	1	2	1
CP	3	1	3
ES	0	2	0
CP+ES	1	2	1

none (0), weak (1), moderate (2), and severe (3).

## Data Availability

Data are contained within the article.
